# DGCR5 Promotes Gallbladder Cancer by Sponging MiR-3619-5p via MEK/ERK1/2 and JNK/p38 MAPK Pathways

**DOI:** 10.7150/jca.46351

**Published:** 2020-07-11

**Authors:** Shilei Liu, Bingfeng Chu, Chen Cai, Xiangsong Wu, Wenyan Yao, Ziyou Wu, Ziyi Yang, Fengnan Li, Yingbin Liu, Ping Dong, Wei Gong

**Affiliations:** 1 Department of General Surgery, Xinhua Hospital, Affiliated to Shanghai Jiao Tong University School of Medicine, No. 1665 Kongjiang Road, Shanghai 200092, China; 2 Shanghai Key Laboratory of Biliary Tract Disease Research, No. 1665 Kongjiang Road, Shanghai 200092, China

**Keywords:** DGCR5, miR-3619-5p, gallbladder cancer, MERK/ERK1/2, JNK/p38 PAPK

## Abstract

Gallbladder cancer (GBC) is a highly aggressive malignant cancer with poor prognosis. Long noncoding RNA (lncRNA) DiGeorge syndrome critical region gene (DGCR5) has been reported to participate in various types of cancers, but its role in GBC remains largely unknown. This study aimed to explore the functions and mechanisms of DGCR5 in GBC. Here, we found that DGCR5 was upregulated in GBC tissues and cell lines. Through functional experiments, it was demonstrated that silence of DGCR5 significantly suppressed the cell proliferation, migration, invasion, and induced apoptosis and cell cycle arrest in GBC cells. In addition, miR-3619-5p was predicted and further verified as the target of DGCR5. Moreover, miR-3619-5p was observed downregulated in GBC tissues and cell lines, and miR-3619-5p mimics repressed the GBC cell proliferation, migration, invasion and could be rescued by DGCR5 overexpression. Mechanistically, it was found that DGCR5 knockdown and miR-3619-5p mimics inactivated the MEK/ERK1/2 and JNK/p38 MAPK pathways. In addition, rescue experiments indicated that inhibition of MEK/ERK1/2 and JNK/p38 MAPK pathways could reverse the effects of DGCR5 overexpression on cell proliferation, migration and invasion. Finally, xenograft model assay was used to validate that knockdown of DGCR5 suppressed GBC via regulating MEK/ERK1/2 and JNK/p38 MAPK pathways *in vivo*. Taken together, it was uncovered in our study that DGCR5 exerts an oncogenic role by sponging miR-3619-5p and activating MEK/ERK1/2 and JNK/p38 MAPK pathways in GBC progression.

## Introduction

Gallbladder cancer (GBC) is the most aggressive and common malignant cancer of the biliary tract, and the 5th commonest gastrointestinal malignancy worldwide 1, 2. So far, the one and only possible curative treatment for GBC is complete surgical resection. However, because of lack of characteristic manifestations at early stage, most GBC patients are diagnosed at an advanced stage, missing the optimal time for treatment. Therefore, GBC is still lethal with a mean survival of only 24.6 months. Besides, the 5-year survival rates of T1 and T2 GBC are 85.9% and 56.1%, but for T3 and T4 GBC the rates slumped to only 19.2% and 14.1% 3-5. Thus, revealing the molecular mechanisms of GBC could be helpful to identify novel diagnostic and therapeutic targets.

Long noncoding RNAs (lncRNAs) are a class of RNAs with longer than 200 nucleotides and without the capacity to code protein. LncRNAs were once considered as transcriptional “noise” but gradually confirmed as vital modulators in several biological processes 6-8, especially in the tumorigenesis and development of cancers 9, 10. Up to now, several lncRNAs have been reported to be involved in the tumorigenesis and progression of GBC. For example, MALAT1 acts as an oncogene that promotes tumor growth and metastasis in GBC through activating the ERK/MAPK pathway 11. LncRNA-PAGBC acts as a ceRNA by sponging microRNA to promote GBC progression via AKT/mTOR pathway 12. However, the overall pathophysiological functions of most other lncRNAs in GBC are still unknown.

LncRNA DiGeorge syndrome critical region gene 5 (DGCR5) was first found in Huntington's disease^13^. Increasing data has displayed that DGCR5 is highly involved in many cancers 14-16. For example, Tang et al. reported that DGCR5 could sponge miR-195 and induce radioresistance in human laryngeal carcinoma cells 14. However, in Chen et al.'s study, DGCR5 exerts anti-cancer effect in papillary throid carcinoma 15. Despite these findings, nothing is known so far about the biological role of DGCR5 in GBC.

Here, we showed that DGCR5 was markedly upregulated whereas miR-3619-5p was downregulated in GBC tissues and cells. DGCR5 knockdown inhibited the GBC cell proliferation, migration, invasion, induced apoptosis, cell cycle arrest* in vitro*, and suppressed GBC tumor growth *in vivo*. Mechanistically, DGCR5 functions as a competing endogenous RNA (ceRNA) by competitively binding to tumor suppressor miR-3619-5p via activating MEK/ERK and JNK/p38 MAPK signaling pathways. So far, this is the first study to highlight the role of DGCR5 in GBC, and to explore the underlying mechanism.

## Materials and Methods

### Patients and specimens

GBC and adjacent non-tumor samples were obtained from 21 pathologically confirmed GBC patients after cholecystectomy, without any radiotherapy or chemotherapy, at the Department of General Surgery, Xinhua hospital, School of Medicine, Shanghai Jiaotong University, China between January 2018 and December 2019. This study was approved by the Ehics Committee of Xinhua Hospital of Shanghai Jiaotong University School of Medicine.

### Cell culture

NOZ, SGC-996, GBC-SD, OCUG and 293T cells were obtained from the Cell Bank of the Type Culture Collection of the Chinese Academy of Sciences (Shanghai, China). NOZ and SGC-996 cells were cultured in Williams' medium and RPMI-1640 medium (Hyclone), respectively. GBC-SD, OCUG and 293T cells were cultured in DMEM (Gibco). All medium was supplemented with 10% FBS (Gibco), 10%, 100U/mL penicillin and 100ug/mL streptomycin (Hyclone). All of the cells were cultured at 37℃ in a 5% CO2 humidified incubator. All cell experiments were conducted within 6 months after obtaining the cells.

### Cell transfection

DGCR5 small interfering RNAs (siRNAs), hsa-miR-3619-5p mimics and inhibitor, and their parental negative control (NC) were synthesized by Genomeditech (Shanghai, China) using RFect reagent (Baidai, China) for transfection according to the manufacturer's protocol. The siRNAs targeting human DGCR5 were: si-DGCR5-1: 5'-GCAAUUAGCUUCAGCUCUAdTdT-3', si-DGCR5-2: 5'-GCGAGAUGUUAUUUCUGAAdTdT-3'. The full-length of DGCR5 overexpression plasmid were synthesized and purchased from Genomeditech (Shanghai, China). ViaFect reagent was used for plasmids transfection following the instructions. Cells were collected after 48 hours transfection. LV-shDGCR5 (5'-GCAAUUAGCUUCAGCUCUAdTdT-3') and the negative control LV-NC using green fluorescence protein (GFP) expressing and puromycin-resistant lentivirus (LV) PGMLV-SC5 vectors were constructed by Genomeditech (Shanghai, China). Lentivirus was used to infect NOZ cells at a multiplicity of infection (MOI) of 90 in medium with 10% FBS. After 48 hours, cells were selected by applying puromycin to construct stable-transfected cells. Transfection efficiency was rough estimated by the expression level of GFP under a fluorescence microscope and further verified by quantitative real-time PCR (qRT-PCR).

### Quantitative real-time PCR

Total RNA was extracted using TRIzol reagent. The cDNAs were generated using the PrimScript Reverse Transcriptase (Takara). The qRT-PCR was performed using SYBR Premix Ex Taq II (Takara) on a StepOnePlus^TM^ Real-time PCR system (Applied Biosystems, USA). The primer sequences are as follows: DGCR5 forward: 5ʹ-ATTTTCCCAGTCTGGCGGAG-3ʹ, reverse: 5ʹ-AGGGCCCCATTATGACTCCT‐3ʹ; miR-3619-5p forward: 5'-TCATCAGCAGGCAGGCTGGTGC-3', reverse 5'-GTGCAGGGTCCCGAGGT-3'; GAPDH forward: 5′-CAACAGCCTCAAGATCATCAGC-3′, reverse: 5′-TTCTAGACGGCAGGTCAGGTC-3′; U6 forward: 5ʹ-CTCGCTTCGGCAGCACA-3ʹ, reverse: 5ʹ-AACGCTTCACGAATTTGCGT-3ʹ. The 2^-ΔΔCT^ method was used to for the relative expression levels.

### CCK-8 assay

The cell viability was detected by CCK-8 assay. Treated cells were plated into a 96-well plate with 2000 cells/well. After 24 hours, each well was added with 10 μL CCK-8 solution and 90 μL complete culture medium, and incubated for 2 hours in dark. Then the absorbance value (OD) at 450 nm was detected using a microplate reader (Bio-Tek).

### Colony forming assay

After transfection for 48 hours, 500 cells were seeded in each well of 6-well plates for 7 to 10 days. The cells were fixed with 4% paraformaldehyde and stained with 0.1% crystal violet (Sigma‐Aldrich). The plates were rinsed, observed and photographed by microscope (Leica).

### Migration and invasion assay

Transwell assays were applied with chamber inserts (Corning, NY, USA) and Corning BioCoat Growth Factor Reduced Matrigel Invasion Chambers (Corning) for migration and invasion assay, respectively. Lower chamber was filled with 750 μL medium containing 10% FBS, and 3 × 10^4^ cells in 200 μL serum-free medium were added in the upper chamber. After 24 hours incubation, cells on the bottom of the lower chambers were fixed with 4% paraformaldehyde and stained with 0.1% crystal violet (Sigma‐Aldrich). Then the chambers were gently washed with PBS, scraped by cotton swabs to remove the cells on the top, and then finally counted under a microscope in 5 random fields of view.

### 5‐Ethynyl‐2′‐deoxyuridine (EdU)-488 proliferation assay and Hoechst 33342 staining

BeyoClick^TM^ EdU-488 proliferation assay (Beyotime, Shanghai, China) was performed according to the manufacture's protocol to detect DNA synthesis. Briefly, cells were incubated with 10 μM EdU for 2 hours. EdU-positive cells were indicated by Azide 488, and Hoechst 33342 was used for cell counterstaining. The cells were viewed and photographed under a fluorescence microscope (Leica).

### Annexin V/PI staining assay for apoptosis

The transfected cells were collected, resuspended with PBS and then stained with 5 μL annexin V-FITC and/or 5 μL propidium iodide (PI) for 30 minutes at room temperature out of light. The cell apoptosis was immediately measured by flow cytometry.

### Cell cycle analysis

Transfected cells were harvested and fixed with 75% ethanol at 4℃ overnight. Then the cells were stained with 10mg/ml RNase and 1mg/ml PI at 37 ℃ for 30 minutes in the dark. The cell cycle was analyzed using flow cytometry.

### Western blot analysis

Total protein was extracted from cells with RIPA lysis buffer. In brief, same amounts of protein samples were separated by 7.5%-15% SDS-PAGE, transferred to PVDF membranes and incubated with specific primary antibodies at 4℃ overnight. Primary antibodies were all purchased from Cell Signaling Technology. Next, the membranes were incubated for 1 hour with secondary antibody, and finally determined by a Gel Doc 2000 (Bio-Rad).

### Bioinformatics analysis

We searched StarBase, DIANA tools and LncRNAdb databases to identify the target miRNAs of DGCR5. MiR-3619-5p, miR-338-3p, miR-137, miR-22-3p and miR-330-5p were selected as the top 5 potential target miRNAs of DGCR5, and we focus our attention on miR-3619-5p for it has the highest predicted scores. The target genes of miR-3619-5p were searched by using DIANA tools, TargetScan and miRDB databases.

### Dual-luciferase activity assay

2 × 10^4^ cells were plated in each well of 24-well plates overnight. Then 293T cells were co-transfected with 10 μg WT (or MUT) DGCR5 reporter plasmids with miR-3619-5p mimics (inhibitors or NC). The relative luciferase activities were determined by Dual-Luciferase Reporter Assay System (Promega) following the instruction.

### Nude mouse subcutaneous xenograft model

Female nude mice (4 weeks, 18-22g) were purchased from the Shanghai Laboratory Animal Center of the Chinese Academy of Sciences (Shanghai, China). Mice were randomly divided into 2 groups and housed in appropriate environment with abundant food and water. NOZ cells infected with LV-NC or LV-shDGCR5 were selected by applying puromycin to construct stable transfected cells. Knockdown efficiency of DGCR5 was estimated by GFP expression intensity and further tested by qRT-PCR. After a week of adjustable feeding, NOZ cells (2 × 10^6^ in 100 μL PBS) transfected with LV-NC or LV-shDGCR5 were subcutaneously injected into the left axilla of the mouse. The tumor volumes were estimated weekly (0.5 × width^2^ × length) by caliper. After 4 weeks, mice were sacrificed by dislocation and the tumors were collected and weighed. Total RNA and proteins were extracted from the 2 tumor groups and the rest of the tumors were stored in 4% paraformaldehyde for further assays. This animal study was approved by the Ethics Committee of Xinhua Hospital Affiliated to Shanghai Jiaotong University School of Medicine.

### Immunohistochemistry

Tumors were fixed in 4% paraformaldehyde, and immunohistochemistry (IHC)was performed following standard process to determine the expression level of Ki-67, PCNA, cleaved-caspase 3, CDK4, p-MEK, p-ERK1/2, p-JNK and p-p38-MAPK. A microscope (Leica) was used to photograph and Image J was used to analyze the IHC results.

### Statistical analysis

All assays were carried out with at least 3 independent experiments. Analyses were performed with GraphPad Prism 7 using student's t test or linear regression when necessary. Data were presented as mean ± standard deviations (SD). P < 0.05 was considered statistically significance.

## Results

### Expression of DGCR5 is upregulated in GBC tissues and cell lines

**Figure 1:**
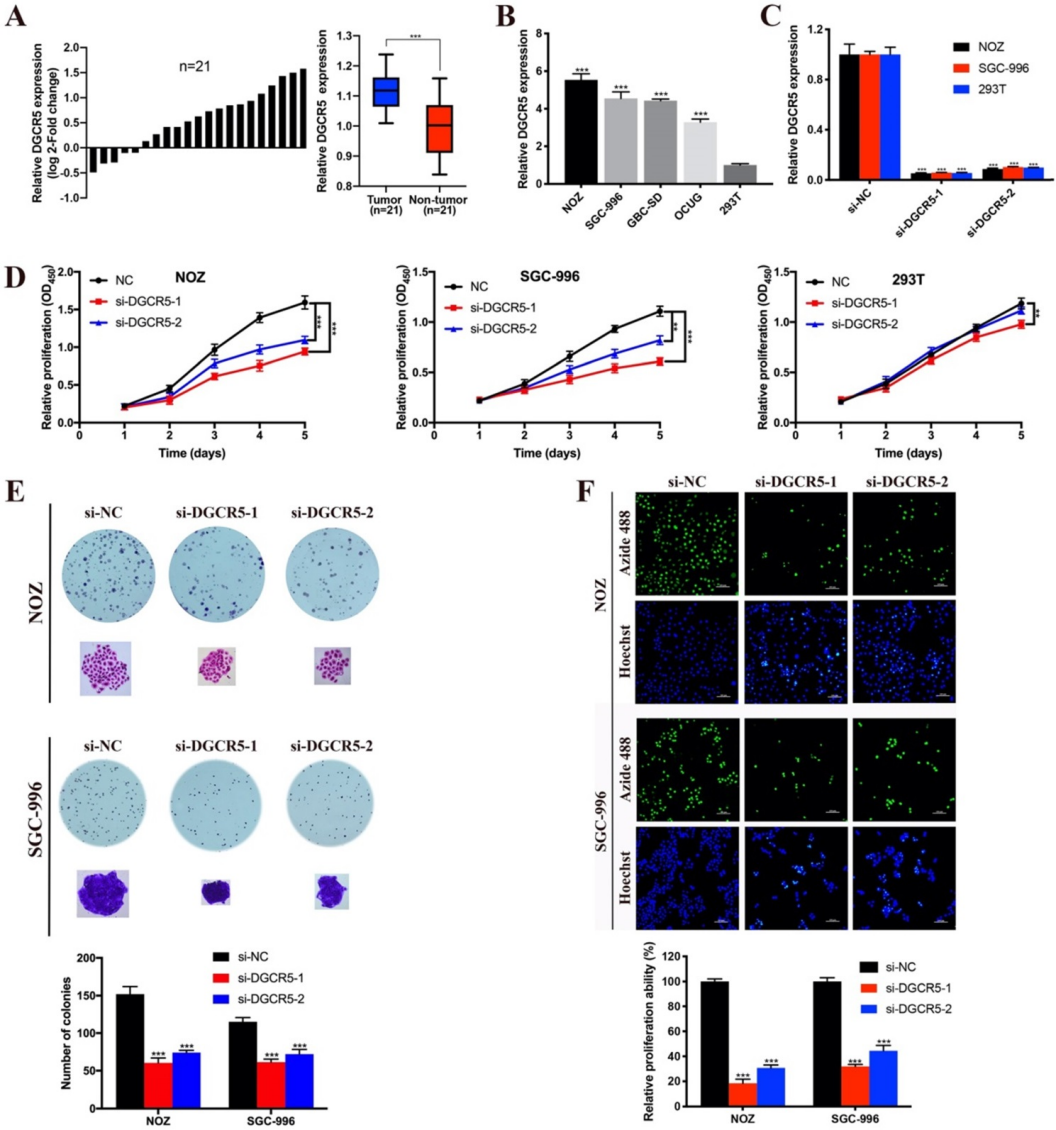
** DGCR5 is upregulated and promotes cell proliferation in GBC. A.** The comparisons of DGCR5 expression levels in 21 paired GBC samples. The results were presented as log 2-fold change of tumor tissues relative to adjacent non-tumor tissues; Relative DGCR5 expression in GBC tissues and adjacent non-tumor tissues. **B.** DGCR5 expression levels in GBC cell lines and 293T cells using qRT-PCR. **C.** Knockdown efficiency of DGCR5 in NOZ, SGC-996 and 293T cells using qRT-PCR. **D.** Cell growth curves of NOZ, SGC-996 and 293T using CCK-8 assay. **E.** DGCR5 knockdown significantly suppressed colony formation of GBC cells. Bar charts show the number of the colonies.** F.** EdU-488 proliferation assay and Hoechst 33342 staining was used to examine cell proliferation. Bar charts show the relative proliferation ability. * P < 0.05, ** P < 0.01, *** P < 0.001.

To detect the expression pattern of DGCR5 in GBC, we performed qRT-PCR to examine the expression levels of DGCR5 in 21 paired GBC patient samples, 4 GBC cell lines and 293T cells. The results showed that DGCR5 expression was higher in GBC tissues compared to adjacent non-tumor tissues (Figure 1A), and also higher in GBC cell lines (NOZ, SGC-996, GBC-SE and OCUG) than 293T cells (Figure 1B) . Then we transfected the NOZ, SGC-996 and 293T cells with DGCR5 siRNAs and further confirmed the knockdown efficiency by qRT-PCR (Figure 1C).

### DGCR5 knockdown inhibits GBC cell proliferation *in vitro*

To investigate whether DGCR5 has effect on GBC cell proliferation, CCK-8 assay, colony formation assay and EdU-488 proliferation assay were conducted. As shown in Figure 1D, the viability of NOZ and SGC-996 cells was notably decreased after DGCR5 knockdown. In addition, DGCR5 depletion significantly reduced the colony formation ability of NOZ and SGC-996 cells (Figure 1E). Consistent with the above results, EdU-488 proliferation assays also demonstrated that knockdown of DGCR5 significantly inhibited the proliferation ability of NOZ and SGC-996 cells (Figure 1F).

### DGCR5 downregulation inhibits migration and invasion of GBC cells

**Figure 2:**
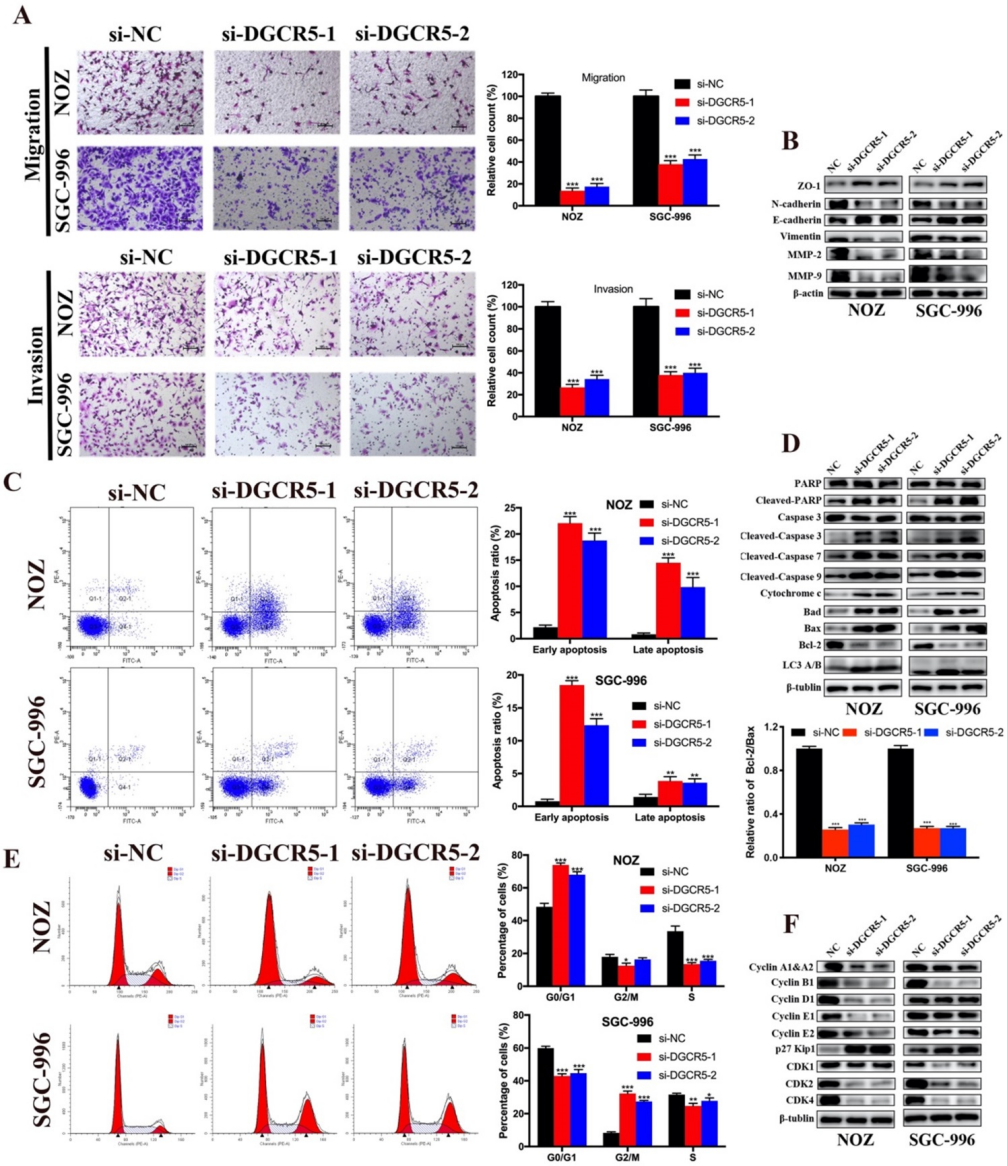
** DGCR5 knockdown inhibited migration and invasion, and induced cell apoptosis and cycle arrest in GBC cells. A.** DGCR5 downregulation notably inhibited the migration and invasion of GBC cells. **B.** EMT-related markers were examined by western blot. **C.** Flow cytometry analysis was conducted to detect the cell apoptosis. The apoptosis ratios are shown in bar charts. **D.** Apoptosis-related markers were detected by western blot. Bcl-2/Bax ratio is showed in bar charts. **E.** Cell cycle was measured by PI staining flow cytometry. The results indicates that G0/G1 arrest in NOZ cells whereas G2/M arrest in SGC-996 cells after DGCR5 knockdown. **F.** The protein level of cell cycle-related markers was detected using western blot. * P < 0.05, ** P < 0.01, *** P < 0.001.

To explore whether DGCR5 expression levels affect GBC metastasis, we applied transwell migration and invasion assays. As shown in Figure 2A, downregulation of DGCR5 significantly inhibited the migration and invasion in GBC cells. To explore whether DGCR5 enhanced the migration and invasion ability of GBC cells through epithelial-mesenchymal transition (EMT) processes, we conducted western blotting to detect the expression of the key biomarkers of EMT. As shown in Figure 2B, after DGCR5 knockdown, the expression levels of ZO-1, E-cadherin were upregulated, whereas the expression levels of N-cadherin, vimentin, MMP-2 and MMP-9 were downregulated. These findings demonstrated that DGCR5 promotes GBC cell migration and invasion by inducing EMT processes.

### Knockdown of DGCR5 induces cell apoptosis and cycle arrest in GBC cells

Flow cytometry assay was carried out to explore whether cell apoptosis and cycle arrest were concerned with the anticancer properties of DGCR5 knockdown. The results showed that knockdown of DGCR5 remarkably increased the ratio of apoptotic cells in GBC cells, and induced G0/G1 arrest in NOZ cells and G2/M arrest in SGC-996 cells (Figure 2C, E). Furtherly, we examined the expression of apoptosis-related and cell cycle-related proteins, and found that downregulation of DGCR5 strongly enhanced the expression of cleaved-PARP, cleaved caspase-3, -7, -9, cytochrome c, Bad and Bax, whereas decreasing the Bcl-2 expression and the ratio of Bcl-2 to Bax. (Figure 2D). LC3 B was found upregulated after DGCR5 knockdown, indicating autophagy might be involved (Figure 2D). The western blot results of cell cycle-related proteins were consistent with the flow cytometry analysis, suggesting G0/G1 arrest in NOZ and G2/M arrest in SGC-996 after DGCR5 depletion (Figure 2F).

### DGCR5 acts as a ceRNA of miR-3619-5p in GBC

**Figure 3:**
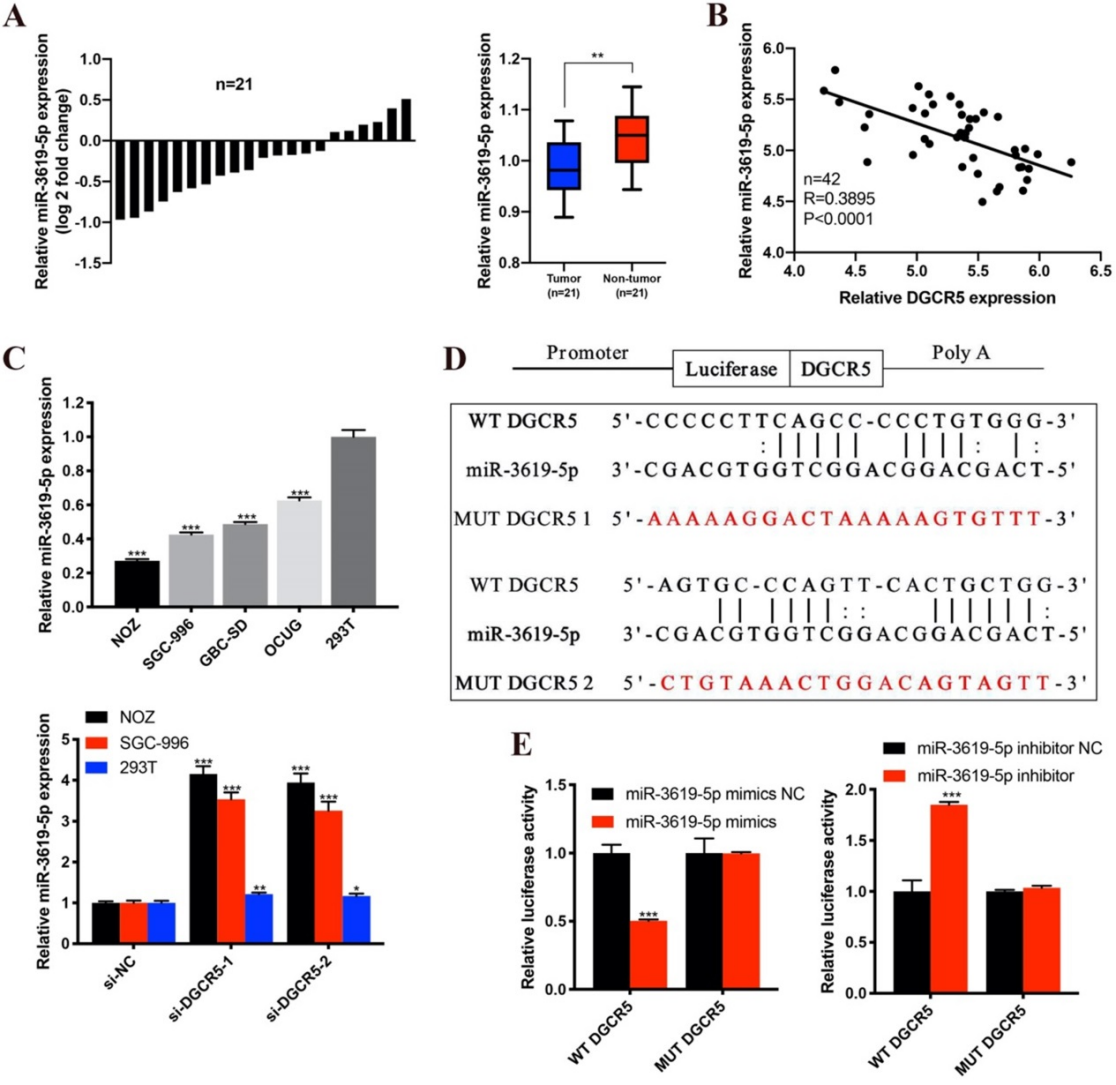
** DGCR5 directly binds to miR-3619-5p. A.** The comparisons of miR-3619-5p expression levels in 21 paired GBC samples. The results were presented as log 2-fold change of tumor tissues relative to adjacent non-tumor tissues; Relative miR-3619-5p expression in GBC tissues and adjacent non-tumor tissues. **B.** The expression of DGCR5 was negatively correlated with miR-3619-5p expression. **C.** Relative miR-3619-5p expression in GBC cell lines and 293T cells; Relative miR-3619-5p expression in NOZ, SGC-996 and 293T cells after DGCR5 knockdown. **D.** The putative binding region of DGCR5/miR-3619-5p. **E.** Dual luciferase reporter assay was performed to verify the direct binding between DGCR5 and miR-3619-5p. * P < 0.05, ** P < 0.01, *** P < 0.001.

Many studies have reported that lncRNAs could function as ceRNA by competitively binding to miRNAs^17^, ^18^. To investigate whether DGCR5 functions as a ceRNA and to search the potential target miRNAs of DGCR5 in GBC, we performed bioinformatics analysis by using StarBase, DIANA tools and LncRNAdb databases. The top 5 potential target miRNAs are as follows: miR-3619-5p, miR-338-3p, miR-137, miR-22-3p and miR-330-5p. Among the potential miRNAs, miR-3619-5p attracted our most attention for it has the highest predicted scores. Moreover, it has been reported that miR-3619-5p could be sponged by lncRNAs and acted as a tumor suppressor in several types of cancers^19^-^21^. Therefore, we detected the miR-3619-5p expression levels in the 21 paired GBC samples and found that, miR-3619-5p expression was downregulated and negative to the DGCR5 expression in GBC tissues (Figure 3A and B). What's more, the miR-3619-5p expression was also downregulated in GBC cell lines compared to 293T cells, and knockdown of DGCR5 increased the expression level of miR-3619-5p (Figure 3C). The putative binding region between DGCR5 and miR-3619-5p was shown in Figure 3D, and the diagrams of the WT DGCR5 and MUT DGCR5 luciferase reporter vector were shown in Figure S1. The results of dual-luciferase reporter assay indicated that the luciferase activity of WT DGCR5 reporter was markedly decreased by miR-3619-5p mimics, whereas was increased by miR-3619-5p inhibitor. Moreover, we mutated the binding sites between miR-3619-5p and DGCR5 and found that the luciferase activity of MUT DGCR5 reporter was not affected by miR-3619-5p mimics or inhibitor (Figure 3E).

### MiR-3619-5p suppresses proliferation, migration and invasion of GBC cells

**Figure 4:**
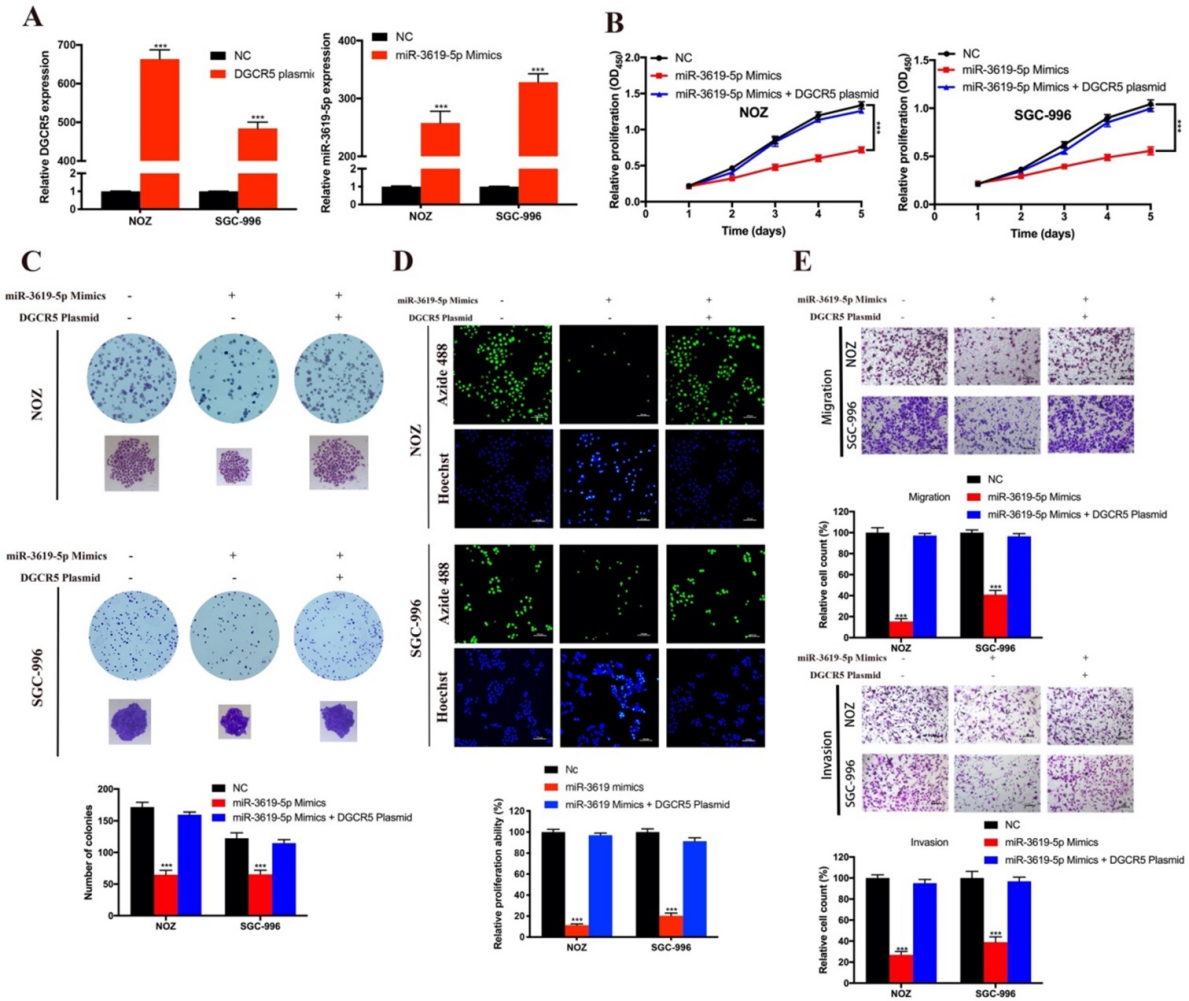
** MiR-3619-5p inhibited GBC cell proliferation, migration and invasion. A.** DGCR5 or miR-3619-5p expression was significantly upregulated in GBC cells after transfected with DGCR5 full length plasmid or miR-3619-5p mimics. **B.** Cell growth curves of NOZ and SGC-996 cells that transfected with miR-3619-5p mimics and/or DGCR5 plasmid. **C.** The microscopic images of colonies formed by transfected GBC cells and the numbers are showed in the bar charts. **D.** EdU-488 assay was applied to examine the proliferation of transfected GBC cells. Bar charts show the relative proliferation ability**. E.** The migration and invasion ability of GBC cells was strongly inhibited by miR-3619-5p mimics, which could be reversed by DGCR5 overexpression. * P < 0.05, ** P < 0.01, *** P < 0.001.

As there is interaction between DGCR5 and miR-3619-5p, we tried to explore the effects of miR-3619-5p on GBC cells. First, we conducted qRT-PCR to examine the DGCR5 and miR-3619-5p levels in NOZ and SGC-996 cells after transfected with full-length DGCR5 plasmid or miR-3619-5p mimics (Figure 4A). Then we performed CCK-8 assay, colony forming assay, EdU-488 proliferation assay and transwell assay. As shown in Figure 4B-E, miR-3619-5p mimics remarkably suppressed the ability of proliferation, migration and invasion in GBC cells. More importantly, DGCR5 overexpression could rescue the effect of miR-3619-5p mimics in GBC cells, suggesting that DGCR5 could sponge miR-3619-5p. These findings demonstrated the tumor suppressor role of miR-3619-5p in GBC.

### DGCR5 downregulation and miR-3619-5p mimics inhibits GBC proliferation, migration and invasion via MEK/ERK1/2 and JNK/p38 MAPK pathways

**Figure 5:**
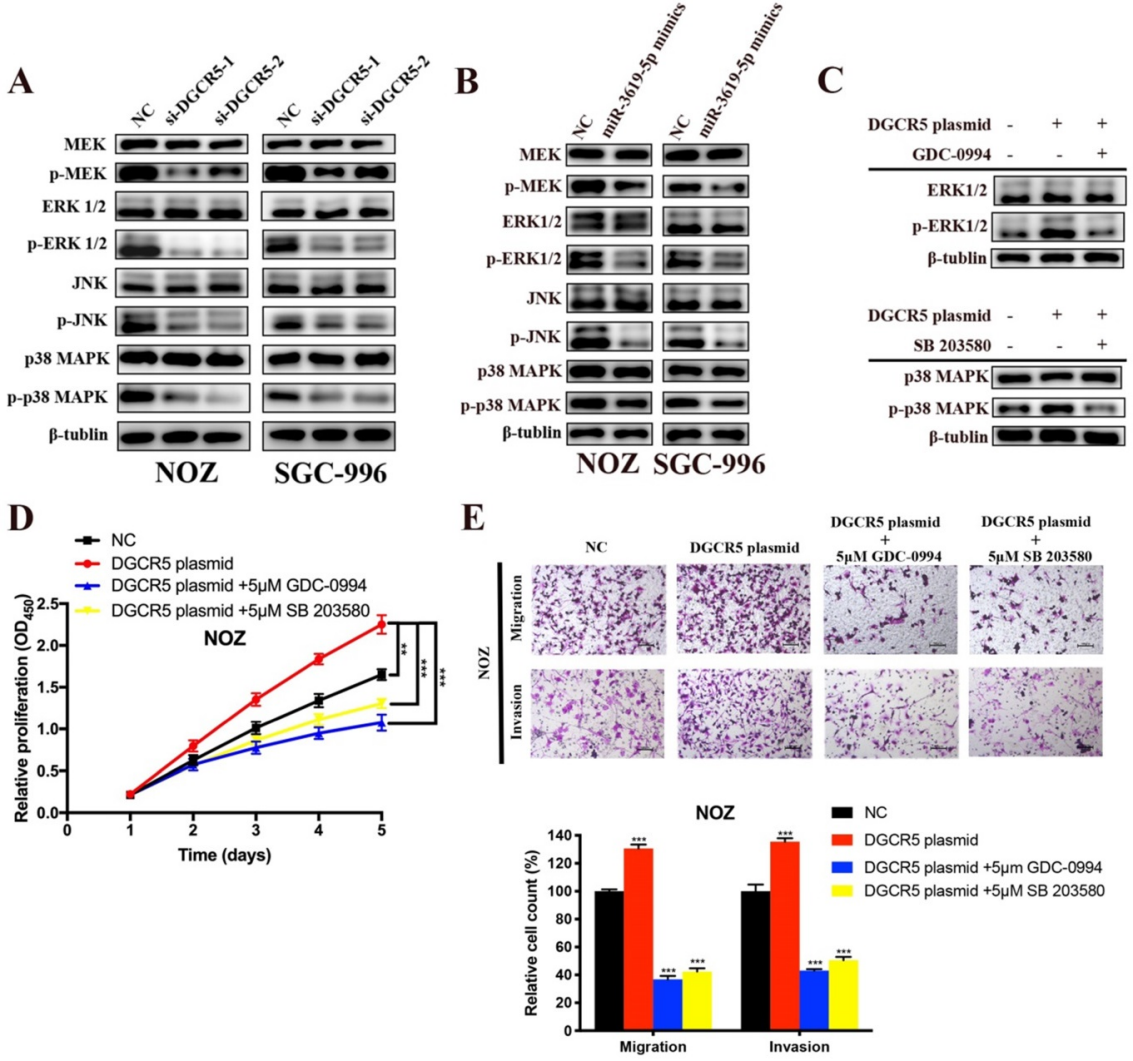
** DGCR5/miR-3619-5p functioned on GBC via modulation of MEK/ ERK1/2 and JNK/p38 MAPK pathways. A-C.** Expression levels of key markers of MEK/ ERK1/2 and JNK/p38 MAPK pathways were tested by western blot. **D and E.** Rescue experiments using CCK-8 assay and transwell assay were conducted to detect the cell proliferation, migration and invasion ability of DGCR5-overexpressed or NC NOZ cells with or without 5μmol/L GDC-0994 (ERK1/2 inhibitor) or SB 203580 (p38 MAPK inhibitor). * P < 0.05, ** P < 0.01, *** P < 0.001.

There is cross-talk between MEK/ERK1/2 and JNK/p38 MAPK pathways 22,23, and these two pathways have been reported to be involved in the progression of GBC^22^-^25^. However, only Wnt/β-catenin pathway has been reported to be associated with DGCR5 or miR-3619-5p^16^, ^26^. Therefore, we investigated whether DGCR5/miR-3619-5p regulated GBC progression via MEK/ERK1/2 and JNK/p38 MAPK pathways. We performed western blotting to examine the protein levels of key regulators of these pathways. As shown in Figure 5A and B, DGCR5 knockdown and miR-3619-5p mimics strongly inhibited the phosphorylation levels of MEK, ERK1/2, JNK and p38 MAPK, and there is no significant change in the total amount of these regulators. Then we undertook rescue experiments to confirm whether MEK/ERK1/2 and JNK/p38 MAPK pathways were involved. NOZ cells were transfected with full-length DGCR5 overexpression plasmid or NC plasmid, and then treated with or without inhibitor against ERK1/2 (GDC-0994) or p38 MAPK (SB 203580) (Figure 5C). The results of CCK-8, migration and invasion assays indicated that either ERK1/2 or p38-MAPK inhibitor could rescue the cell proliferation, migration and invasion that enhanced by DGCR5 overexpression (Figure 5D and E).

### DGCR5 knockdown suppresses the growth of GBC *in vivo*

**Figure 6:**
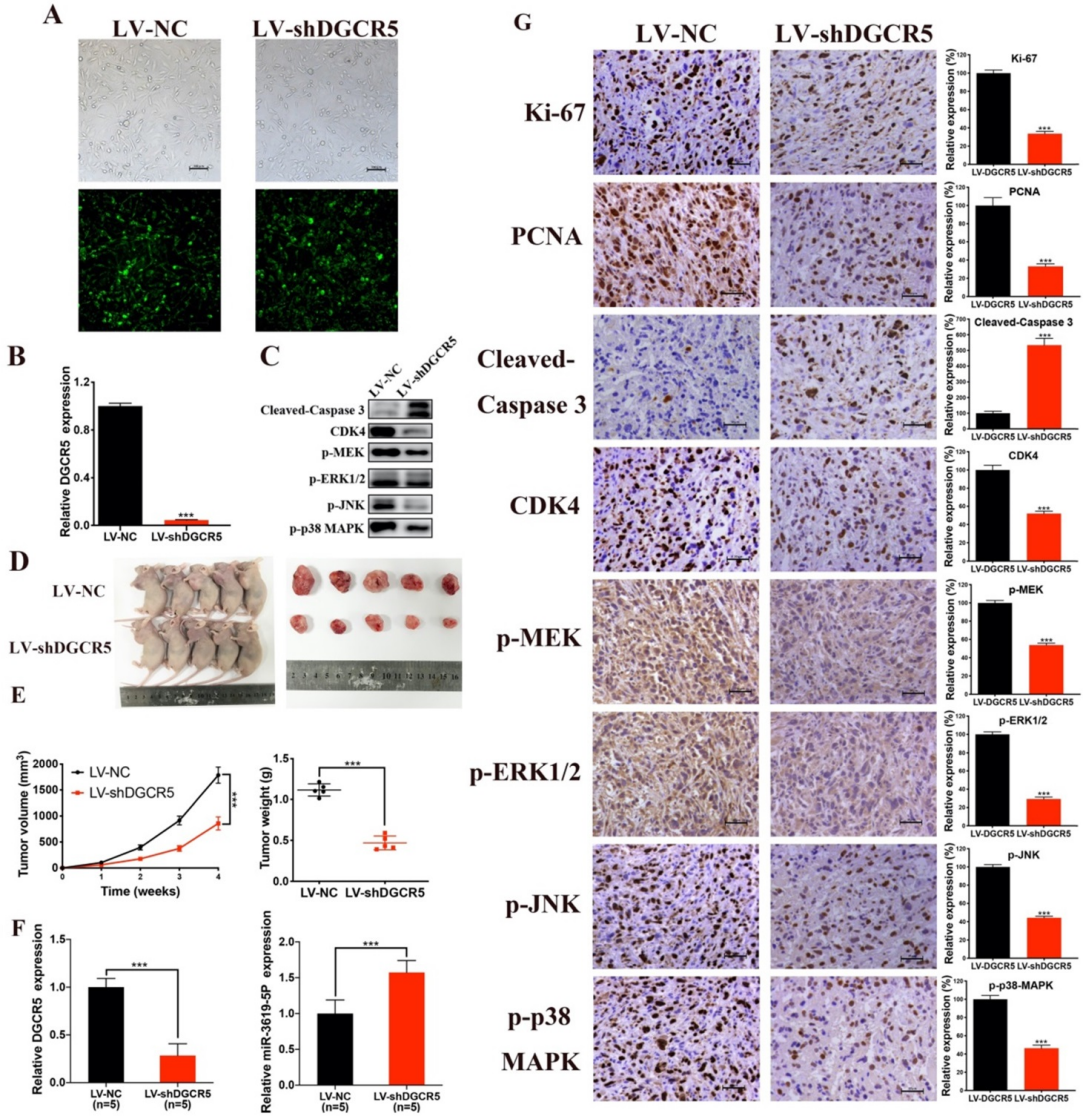
** DGCR5 knockdown significantly suppressed tumor growth *in vivo*. A and B.** The lentivirus transfection efficiency was estimated by the expression level of GFP under a fluorescence microscope and was verified by qRT-PCR. **C.** Proteins were exacted from the tumors and using western blot to measure cleaved- caspase 3, CDK4, p-MEK, p-ERK1/2, p-JNK and p-p38 MAPK expression. **D.** The tumors of LV-shDGCR5 group were smaller than that of LV-NC group. **E.** The tumor volumes and weights in LV-NC and LV-shDGCR5 xenografts. **F.** The relative DGCR5 and miR-3619-5p expression in the LV-NC and LV-shDGCR5 xenografts. **G.** IHC staining assay was conducted to detect the expression of the key markers in tumor tissue, the relative expression are showed in the bar charts. * P < 0.05, ** P < 0.01, *** P < 0.001.

The effect of DGCR5 on GBC growth *in vivo* was then investigated. We first constructed NOZ cells that stable transfected with LV-NC or LV-shDGCR5. Transfection efficiency of lentivirus was estimated by the GFP expression level under a fluorescence microscope and further confirmed by qRT-PCR (Figure 6A and B). Then we undertook xenograft growth assays by injecting the LV-shDGCR5 or LV-NC NOZ cells into nude mice. Compared with negative control, the tumor volume and weight of LV-shDGCR5 xenografts was remarkably suppressed (Figure 6D-E). Compared with the LV-NC xenografts, the expression level of DGCR5 was lower whereas that of miR-3619-5p was higher in LV-shDGCR5 xenografts (Figure 6F). In addition, DGCR5 downregulation markedly suppressed the levels of Ki-67, PCNA, CDK4, p-MEK, p-ERK1/2, p-JNK and p-p38 MAPK, while increased the cleaved-caspase 3 expression (Figure 6C and G). Taken together, these results characterized the essential role of DGCR5 in the tumorigenesis of GBC cells *in vivo*.

## Discussion

Amounting evidence has been shown that lncRNA DGCR5 is involved in various cancers, however, the effect of DGCR5 in the tumorigenesis still remains controversial. Our study is so far the first research of the DGCR5 expression pattern and biological function in GBC. We found that DGCR5 was significantly overexpressed and acted as an oncogene in GBC, in contrast, miR-3619-5p was downregulated and acted as a tumor suppressor in GBC. Mechanistically, DGCR5 inhibits GBC cell proliferation, migration, invasion and tumor growth via sponging miR-3619-5p and activating MEK/ERK1/2 and JNK/p38 MAPK pathways.

GBC is characterized by strong invasion and metastasis to distant organs, ultimately resulting in poor prognosis 27,28. During metastatic progression, cancer cells were endowed with enhanced metastasis property by EMT process 29. To investigate whether DGCR5 affected the metastasis and EMT process of GBC, we performed transwell assays and detected the protein level of EMT markers. The results showed that DGCR5 knockdown significantly inhibited migration and invasion ability of GBC cells. What's more, DGCR5 also affected the expression levels of EMT biomarkers. Knockdown of DGCR5 increased the expression of ZO-1, E-cadherin and decreased the expression of N-cadherin, Vimentin, MMP-2 and -9. These data demonstrated the important function of DGCR5 in the migration, invasion and EMT process of GBC cells, suggesting the potential role of DGCR5 in mediating GBC metastasis.

Apoptosis is a vital cell process also called programmed cell death, the modulation of which is associated with a variety of diseases, including cancer 30. Apoptosis can be activated via several pathways. In the intrinsic pathway (mitochondrial pathway), cytochrome c and apoptosomes induced autocatalysis and activated by cleaving the caspase-9 , caspase-7 and caspase-3 31, 32. Caspase-3 is the key executioner leading to apoptosis by cleaving multiple vital cellular substrates such as PARP^33^. In addition, the intrinsic pathway is mainly modulated by BCL-2 family, including Bad, Bax and BCL-2 31. We observed that DGCR5 silencing strongly induced apoptosis and increased the levels of cleaved PARP, cleaved caspase -3, -7, -9, cytochrome c, Bad, Bax, and decreased the BCL-2 expression and particularly the BCL-2/Bax ratio. These data suggested that DGCR5 knockdown induced apoptosis in GBC via the intrinsic pathway. Moreover, the increased expression level of LC3 B indicated that autophagy activation may have occurred by DGCR5 silencing.

Normal tissue homeostasis and development is largely determined by the precise regulation of cell cycle, for the dysregulation of which may lead to cell loss or excess proliferation that ultimately resulting in the formation of cancer 34. We conducted flow cytometry to explore the role of DGCR5 on the cell cycle of GBC. The knockdown of DGCR5 was found to significantly increase the proportion of G0/G1 in NOZ cells whereas G2/M in SGC-996 cells. Then we used western blotting to detect the protein levels of the key cell cycle regulators, including cyclin-A1&A2, -B1, -D1, -E1, -E2, p27 Kip1, CDK1, CDK2 and CDK4, their alteration was consistent with the flow cytometry analysis. Together, the above results suggested that knockdown of DGCR5 induced cell cycle arrest in GBC cells.

LncRNAs may function through various mechanisms, ceRNA theory has increasingly emerged as a regulatory mechanism between lncRNAs and miRNAs, indicating that lncRNAs function as miRNA sponges to negatively regulating the expression level of miRNA.^35^. After bioinformatics prediction, we identified miR-3619-5p may be a target miRNA of DGCR5, and their direct interaction was confirmed by dual-luciferase assay. Previous studies have revealed that miR-3619-5p plays a tumor suppressive role in various cancers 19, 36, 37. Then we conducted a series of loss/gain-of-function assays to explore the expression pattern and biological role of miR-3619-5p in GBC. In our research, we have found that the expression of miR-3619-5p was downregulated in GBC tissues and cell lines, and was inversely proportional to the expression of DGCR5. In addition, miR-3619-5p mimics exerted anti-tumor effect that inhibiting the cell proliferation, migration and invasion of GBC. What's more, DGCR5 overexpression could rescue the effects of miR-3619-5p mimics. Collectively, these data demonstrated that DGCR5 acts as a ceRNA of miR-3619-5p and inhibited the tumor suppressive effects of miR-3619-5p in GBC.

MEK/ ERK1/2 and JNK/p38 MAPK pathways, between which there are intricate cross-talk, are closely related to the tumorigenesis of GBC 25, 33, 38, 39. Therefore, we investigated whether DGCR5/miR-3619-5p functioned on GBC via the modulation of MEK/ ERK1/2 and JNK/p38 MAPK pathways. It was found that DGCR5 knockdown and miR-3619-5p mimics notably depleted the phosphorylation levels of MEK, ERK1/2, JNK and p38 MAPK. What's more, rescue experiments using ERK1/2 inhibitor (GDC-0994) or p38 MAPK inhibitor (SB 203580) could reverse the cell proliferation, migration and invasion enhanced by DGCR5 overexpression. The above results indicated that DGCR5/miR-3619-5p promotes GBC progression by activating MEK/ ERK1/2 and JNK/p38 MAPK pathways.

Previous studies have revealed that lncRNAs could exert its function by sponging miRNA to regulate gene expression^18^, ^40^, ^41^. So far, our study has demonstrated that DGCR5 affected several cellular functions including EMT process, cell apoptosis and cycle via activating the MEK/ ERK1/2 and JNK/p38 MAPK pathways. Therefore, we performed bioinformatic analysis to tentatively explore the potential genes of DGCR5/miR-3619-5p axis. Among the genes that were hit by this axis we have identified, BAX, CDK2, ERK1, ERK2 and JNK were predicted to be the target genes of miR-3619-5p. However, further research is needed to confirm these predictions.

In summary, our study characterized the role of DGCR5 in GBC for the first time, demonstrating that DGCR5 promotes GBC cell proliferation, migration and invasion by sponging miR-3619-5p via activating the MEK/ ERK1/2 and JNK/p38 MAPK pathways. These results underscore the importance of DGCR5/ miR-3619-5p/ MEK/ ERK1/2 and JNK/p38 MAPK axis in GBC. Our study provides the first evidence that DGCR5/miR-3619-5p network may be a novel biomarker for early diagnosis and treatment of GBC.

## Supplementary Material

Supplementary figure.

## Abbreviations

GBC: gallbladder cancer
LncRNA: Long noncoding RNA
DGCR5: DiGeorge syndrome critical region gene 5
miRNA: microRNA
ceRNA: competing endogenous RNA
siRNA: small interfering RNA
NC: negative control
GFP: green fluorescence protein
LV: lentivirus
MOI: multiplicity of infection
qRT-PCR: quantitative real-time PCR
EdU: 5-Ethynyl-2′-deoxyuridine
PI: propidium iodide
IHC: Immunohistochemistry
SD: standard deviations
EMT: epithelial-mesenchymal transition
