# DGCR5 Promotes Gallbladder Cancer by Sponging MiR-3619-5p via MEK/ERK1/2 and JNK/p38 MAPK Pathways: Erratum

**DOI:** 10.7150/jca.106439

**Published:** 2025-01-01

**Authors:** Shilei Liu, Bingfeng Chu, Chen Cai, Xiangsong Wu, Wenyan Yao, Ziyou Wu, Ziyi Yang, Fengnan Li, Yingbin Liu, Ping Dong, Wei Gong

**Affiliations:** 1 Department of General Surgery, Xinhua Hospital, Affiliated to Shanghai Jiao Tong University School of Medicine, No. 1665 Kongjiang Road, Shanghai 200092, China; 2 Shanghai Key Laboratory of Biliary Tract Disease Research, No. 1665 Kongjiang Road, Shanghai 200092, China

We recently found that there is an inadvertent mistake in our article entitled “DGCR5 Promotes Gallbladder Cancer by Sponging MiR-3619-5p via MEK/ERK1/2 and JNK/p38 MAPK Pathways”, due to our carelessness. In the article, the representative image of 'p-JNK' for the 'LV-NC' group in Figure 6G was inadvertently misplaced during the image editing process. We would like to correct this with the appropriate image in Figure 6G below. The correction does not affect the original results and conclusions. We are sorry for this mistake and apologize for any inconvenience caused.

## Figures and Tables

**Figure 6:**
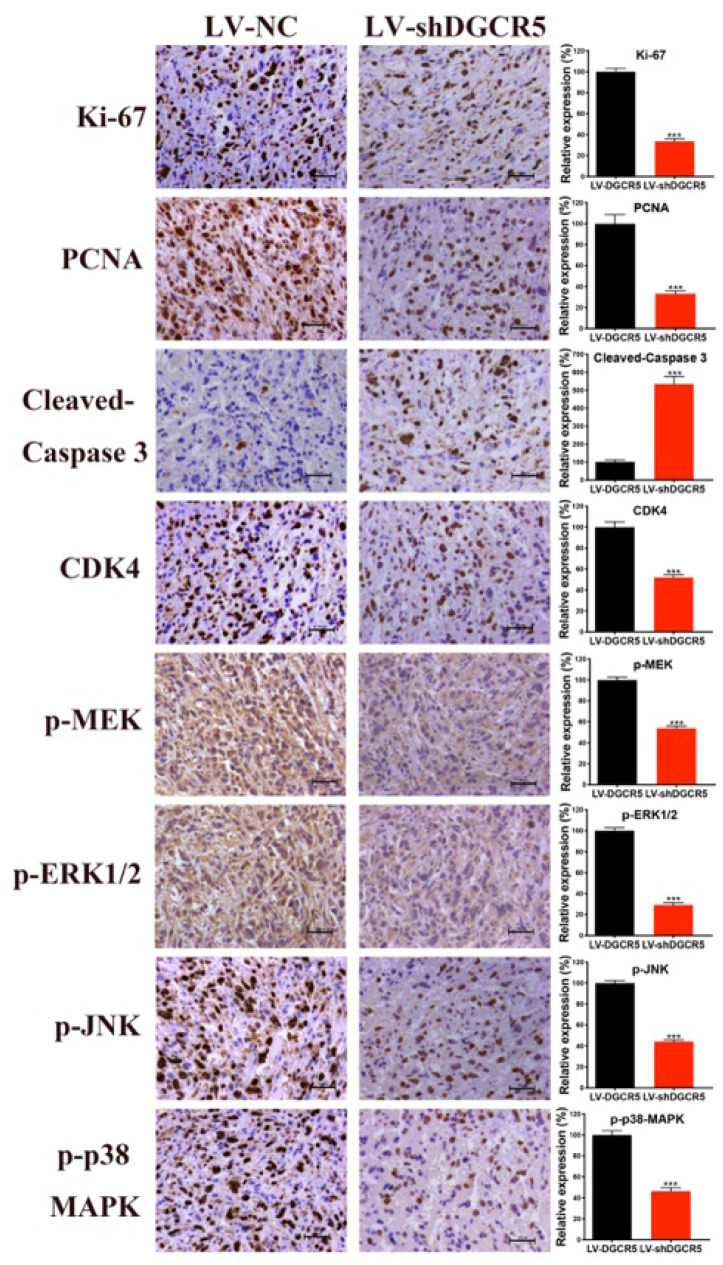
** DGCR5 knockdown significantly suppressed tumor growth *in vivo*.** G. IHC staining assay was conducted to detect the expression of the key markers in tumor tissue, the relative expression is showed in the bar charts. * P < 0.05, ** P < 0.01, *** P < 0.001.

