# Supplementary figures and images for: DGCR5 Promotes Gallbladder Cancer by Sponging MiR-3619-5p via MEK/ERK1/2 and JNK/p38 MAPK Pathways
Source: J Cancer. 2020 Jul 11;11(18):5466–77. doi: 10.7150/jca.46351 (PMC7391188; doi:10.7150/jca.46351)

**Figure S1. Diagrams of WT DGCR5 and MUT DGCR5 luciferase reporter vector.**

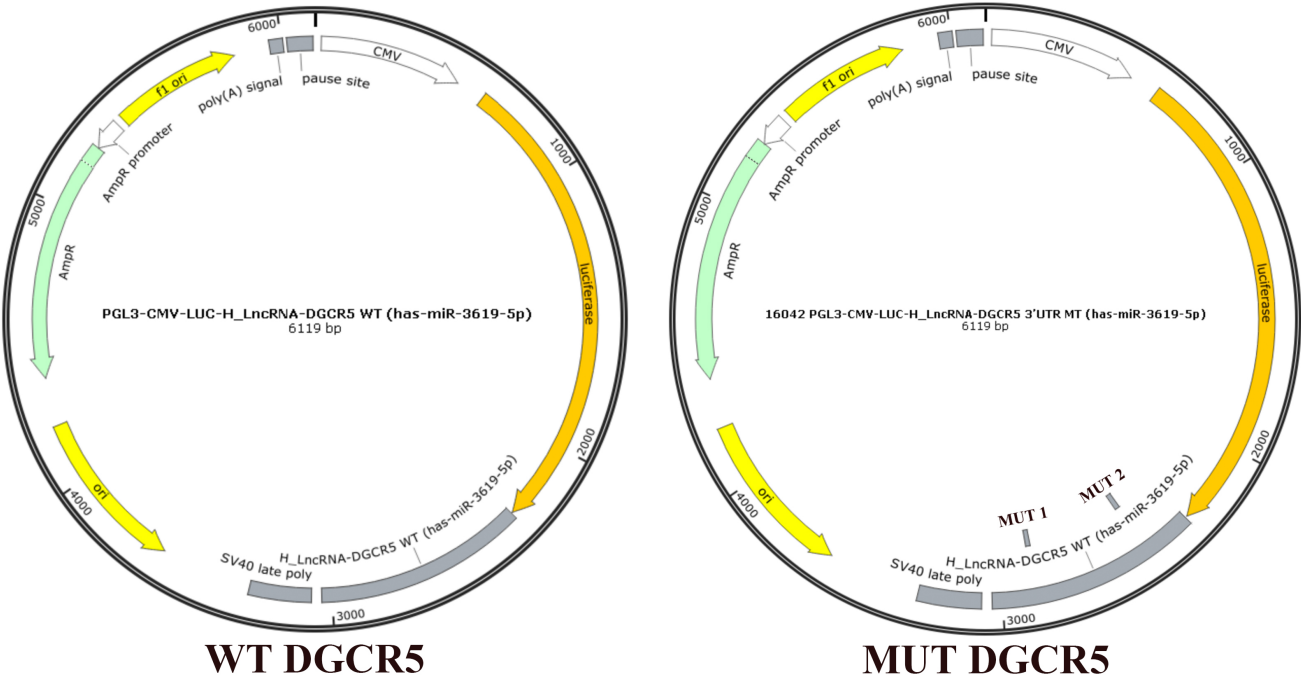

Supplement: Supplementary file 1 — Supplementary figure. [file jcav11p5466s1.pdf]
